# Thermodynamic Assessment of Bio-Oriented Ti-Ta-Sn System

**DOI:** 10.3390/ma14061568

**Published:** 2021-03-23

**Authors:** Lifang Yan, Yingbiao Peng, Tao Li, Lianwu Yan, Shiwen He, Tao Xu

**Affiliations:** 1 College of Metallurgical and Materials Engineering, Hunan University of Technology, Zhuzhou 412007, China; lifang_yan_hut@163.com (L.Y.); taoli_hut@163.com (T.L.); yanlianwu2008@126.com (L.Y.); hswcsu@126.com (S.H.); 2 State Key Laboratory of Cemented Carbide, Zhuzhou Cemented Carbide Group Co., LTD., Zhuzhou 412000, China; xutao2005@126.com; 3 State Key Laboratory for Powder Metallurgy, Central South University, Changsha 410083, China

**Keywords:** Ti-Ta-Sn ternary system, phase diagram, thermodynamic modeling, thermodynamic predictions

## Abstract

The alloying elements Ta and Sn can effectively increase the stability of β-bcc phase, reduce Young’s modulus and improve the shape-memory property of Ti-based biomedical alloys. The development of the thermodynamic database for Ti-based biomedical alloys promises thermodynamic predictions in composition design and process optimization. In this work, one key sub-ternary Ti-Ta-Sn system has been thermodynamically assessed based on critical evaluation of experimental phase equilibria. A self-consistent thermodynamic description for the Ti-Ta-Sn system including one ternary compound Ti_36_Ta_28_Sn_36_ and six binary compounds considering the solubility of the third element has been obtained. Two isothermal sections at 973 and 1173 K and the liquidus projection have been calculated. Comparisons between the calculated and experimental phase equilibria validate the reliability of the present thermodynamic description. The influence of Ta and Sn contents on the transformation temperature and amount of α_hcp-Ti phase in β_bcc-(Ti,Ta) phase has been investigated based on thermodynamic calculations. The solidified phases in Ti-20Ta-*x*Sn (*x* = 5, 15 and 25 at.%) as-cast alloys have been thermodynamically calculated based on Scheil solidification simulations. The presently developed thermodynamic description of the Ti-Ta-Sn system would promote the establishment of muti-component Ti-based thermodynamic database and guide the development of Ti-based alloys.

## 1. Introduction

Metastable β_type Ti-based alloys have been widely used in biomedical applications because of their desirable properties such as similar Young’s modulus to human bones, high strength, corrosion resistance behavior and good biocompatibility [1,2,3]. However, the presence of α” martensite transformed from β during quenching or deformation significantly enlarges the elastic moduli difference between implant biomaterials and human bone [3]. In order to improve the biomedical properties of Ti-based alloys, alloying elements like Ta, Nb, Sn, Zr, Mo, etc have been reported to promise required properties [4,5,6]. It is reported that the alloying of Ta can effectively reduce the martensitic transformation temperature and thus stabilize β_bcc phase [2,3,4,5]. The third alloying element of Sn in Ti-Ta-based alloys can further adjust their properties, such as reducing elastic moduli, enhancing strength, improving corrosion resistance and so on [7,8]. To some extent, the mechanical properties of a material are related to its microstructure. As a result, it is of great importance to understand the thermodynamic behavior of the phases during the development of materials.

Generally, metastable β_bcc phase can be retained by rapid cooling. In order to obtain optimal combination of properties, Ti-based alloys composed with metastable β_bcc phase can be subjected to aging treatment, and subsequently precipitating α_hcp phase [9,10]. For pure Ti, an allotropic transformation from β_bcc phase to α_hcp phase occurs at about 880 °C during cooling. For multicomponent Ti-based alloys, this transformation temperature strongly depends on alloy composition. Thus, an optimal combination of properties corresponding to the strengthening precipitates could be obtained through the design of alloying composition and aging heat treatment. CALPHAD technique has been proven to be a powerful tool to analysis phase stabilities and phase transformations [11], which can provide a strong theoretical basis to the development of Ti based alloys. However, the thermodynamic description of the Ti-Ta-Sn system has not been reported yet.

Hence, the present work aims to thermodynamically model the Ti-Ta-Sn system based on critical evaluation of experimental phase equilibria in the literature, to study the influence of Ta and Sn content on the phase transformation between α_hcp-Ti and β_bcc-(Ti,Ta) phases.

## 2. Experimental Information

The symbols denoting the phases in the Ti-Ta-Sn system and the crystal structure information are listed in Table 1 for facilitating reading.

**Figure 1:**
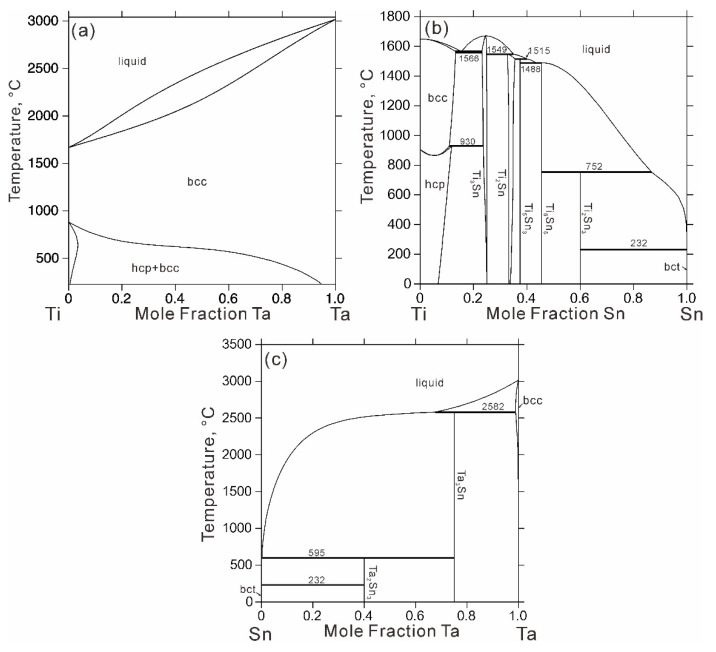
Calculated binary phase diagrams: (**a**) Ti-Ta data from Ref. [12], (**b**) Ti-Sn data from Ref. [13] and (**c**) Ta-Sn data from Ref. [14].

The literature information for the sub-binary systems have been critically reviewed in previous works [12,13,14,15,16,17], which will not be repeated here. The recent evaluations of the Ti-Ta [12], Ti-Sn [13] and Ta-Sn [14] binary systems are considered to be reliable and adopted in the present assessment of the Ti-Ta-Sn ternary system. Figure 1 shows the calculated phase diagrams of the Ti-Ta, Ti-Sn and Ta-Sn systems.

The major contribution to the phase diagram of the Ti-Ta-Sn system comes from the contributions by Wang et al. [16,17]. Using X-ray diffraction (XRD), scanning electron microscopy (SEM) and electron probe microanalysis (EPMA) techniques, the isothermal sections of the Ti-Ta-Sn ternary system were constructed based on 12 equilibrium alloys at 973 K [16] and 11 equilibrium alloys at 1173 K [17]. A total of four two-phase regions and three three-phase regions at 973 K, and five two-phase regions and two three-phase regions at 1173 K were experimentally detected [16,17]. One stoichiometric ternary compound was detected in the Ti-Ta-Sn system, Ti_36_Ta_28_Sn_36_, at 1173 K [17]. No crystallographic information of Ti_36_Ta_28_Sn_36_ has been reported in the literature. The sole study on this ternary compound was conducted by Wang et al. [17]. However, they only determined its composition using EPMA without further studying its crystal structure. Additionally, the solubility of the third element in boundary compounds were experimentally determined by means of EPMA technique [16,17]. The solubilities of Ta were determined to be 8.3 at.% at 973 K and 9.3 at.% at 1173 K in Ti_3_Sn, no less than 8.8 at.% at 973 K and no less than 7.3 at.% at 1173 K in Ti_2_Sn, about 4.2 at.% at 973 K and 6.1 at.% at 1173 K in Ti_5_Sn_3_, about 16.8 at.% at 973 K and no less than 15.5 at.% at 1173 K in Ti_6_Sn_5_, and no less than 15.8 at.% at 973 K in Ti_2_Sn_3_ [16,17]. The EPMA results indicated that Ta mainly replaces Ti in all the Ti-Sn boundary compounds and Ti mainly replaces Ta in both Ta_3_Sn and Ta_2_Sn_3_.

## 3. Thermodynamic Models

In the present modeling, the Gibbs energy expressions for pure Ti, Ta and Sn are taken from the SGTE compilation by Dinsdale [18]. For the Ti-Ta binary system, the thermodynamic description from Saunders [12] have been have been applied in the development of thermodynamic database for cemented carbides [11,19], which was considered to be reliable and thus adopted in the present work. The first assessment of the Ti-Sn binary system is from the work by Hayes [20]. However, the later detected intermetallic compound, Ti_2_Sn_3_, was not considered in that work. Recently, another two reassessments of the Ti-Sn system were reported [13,21], which show no major difference in the reproduction of experimental data. The more recent assessment from Yin et al. [13] performed based on their own experimental data at the Sn-rich side was thus adopted in the present work. To date, there are two assessments for the Ta-Sn binary system in the literature [14,15]. Two intermetallic compounds, Ta_3_Sn and Ta_2_Sn_3_, were experimentally detected by Basile [22] and were further accepted and used to construct the Ta-Sn phase diagram by Okamoto [23]. However, Ta_2_Sn_3_ was simply modeled as TaSn_2_ in the assessment by Marker et al. [15] in order to keep compatible towards similar systems like V-Sn and Nb-Sn. The treatment of TaSn_2_ ignoring its experimental composition seems to be unsuitable, especially when regarding the Ti_2_Sn_3_ compound in another similar Ti-Sn system. Consequently, the assessment of the Ta-Sn system from Wang et al. [14] was adopted in the present work.

The phases in the Ti-Ta-Sn system were modeled according to compound energy formalism [24]. The present modeling of the Ti-Ta-Sn system was based on the recent evaluations of the Ti-Ta [12], Ti-Sn [13] and Ta-Sn [14] binary systems. The solution phases, liquid, bcc, hcp, and bct, were described by substitutional solution model using the Redlich-Kister polynominal [25]:

(1) $${\;}^{0}G_{m}^{\varphi }=\sum_{i}x_{i}{\;}^{0}G_{i}^{\varphi }+RT\sum_{i}x_{i}\ln x_{i}+\sum_{i,j>i}x_{i}x_{j}\sum_{v}{\;}^{v}L_{i,j}^{\varphi }{\left(x_{i}-x_{j}\right)}^{v}+{\;}^{\mathrm{ex}}G_{m}^{\varphi }$$

(2) $${\;}^{\mathrm{ex}}G_{m}^{\varphi }=x_{\mathrm{Sn}}x_{\mathrm{Ta}}x_{\mathrm{Ti}}(x_{\mathrm{Sn}}{\;}^{0}L_{\mathrm{Sn},\mathrm{Ta},\mathrm{Ti}}^{\varphi }+x_{\mathrm{Ta}}{\;}^{1}L_{\mathrm{Sn},\mathrm{Ta},\mathrm{Ti}}^{\varphi }+x_{\mathrm{Ti}}{\;}^{2}L_{\mathrm{Sn},\mathrm{Ta},\mathrm{Ti}}^{\varphi })$$

where $x_{i}$ and $x_{j}$ represent the molar fractions of elements (*i, j* = Sn, Ta, Ti). ${\;}^{0}G_{i}^{\varphi }$ is the reference Gibbs energy of pure *i* at 298 K and 1 bar. ${\;}^{v}L_{i,j}^{\varphi }$ is the interaction parameter from binary system. ${\;}^{v}L_{\mathrm{Sn},\mathrm{Ta},\mathrm{Ti}}^{\varphi }$(*v* = 0, 1, 2) are ternary interaction parameters to be evaluated in the present work.

According to the sublattice model developed by Hillert and Staffansson [26], a two-sublattice model was employed to describe the intermetallic binary compounds, Ti_3_Sn, Ti_2_Sn, Ti_5_Sn_3_, Ti_6_Sn_5_, Ti_2_Sn_3_, Ta_3_Sn, Ta_2_Sn_3_, and a three-sublattice model for stoichiometric ternary compound Ti_36_Ta_28_Sn_36_. Taking a phase ϕ modeled by (Sn, Ta, Ti)*_a_*(Sn, Ta, Ti)*_c_* for example, its Gibbs energy can be expressed as follows:

(3) $${\;}^{0}G_{m}^{\phi }=\sum_{i}\sum_{j}{y^{\prime }}_{i}{y^{\prime \prime }}_{j}G_{i:j}^{\phi }+RT(a\sum_{i}{y^{\prime }}_{i}\ln{y^{\prime }}_{i}+c\sum_{j}{y^{\prime \prime }}_{j}\ln{y^{\prime \prime }}_{j})+{\;}^{\mathrm{ex}}G_{m}^{\phi }$$

(4) $${\;}^{\mathrm{ex}}G_{m}^{\phi }=\sum_{i}{y^{\prime }}_{i}\sum_{m,n>m}{y^{\prime \prime }}_{m}{y^{\prime \prime }}_{n}\sum_{v}{\;}^{v}L_{i:m,n}^{\phi }{\left({y^{\prime \prime }}_{m}-{y^{\prime \prime }}_{n}\right)}^{v}+\sum_{i,j>i}{y^{\prime }}_{i}{y^{\prime }}_{j}\sum_{m}{y^{\prime \prime }}_{m}\sum_{v}{\;}^{v}L_{i,j:m}^{\phi }{\left({y^{\prime \prime }}_{m}-{y^{\prime \prime }}_{n}\right)}^{v}+\sum_{i,j}\sum_{m,n}{y^{\prime }}_{i}{y^{\prime }}_{j}{y^{\prime \prime }}_{m}{y^{\prime \prime }}_{n}L^{rec}$$

where *y*′ and *y*″ are the site fractions of element in the first and second sublattices. $G_{i:j}^{\phi }$ indicates the Gibbs energy of a hypothetical state where the first and second sublattices are completely filled by *i* and *j*, respectively. The second term of Equation (3) describes the energy contribution due to ideal mixing in each sublattice. $L^{rec}$ represents the reciprocal parameter.

Using Thermo-Calc packages [27], the model parameters were evaluated according to the uncertainty of adopted experimental data and changed by trial and error during the assessment, until most of the selected experimental information can be reproduced within the expected uncertainty limits. The thermodynamic parameters obtained in the present work are listed in Table 2.

**Table 2:** Summary of the thermodynamic parameters in the Ti–Ta–Sn system ^★^.

Phase/Model	Thermodynamic Parameters	Reference
Liquid: (Sn,Ta,Ti)_1_	${\;}^{0}L_{\mathrm{Sn},\mathrm{Ta}}^{\mathrm{liquid}}=-9735.8+17.37T$	[14]
	${\;}^{0}L_{\mathrm{Ta},\mathrm{Ti}}^{\mathrm{liquid}}=1000$	[12]
	${\;}^{1}L_{\mathrm{Ta},\mathrm{Ti}}^{\mathrm{liquid}}=-7000$	[12]
	${\;}^{0}L_{\mathrm{Sn},\mathrm{Ti}}^{\mathrm{liquid}}=-91,598-0.9416T$	[13]
	${\;}^{1}L_{\mathrm{Sn},\mathrm{Ti}}^{\mathrm{liquid}}=45,682-12.1045T$	[13]
bcc: (Sn,Ta,Ti)_1_(Va)_3_	${\;}^{0}L_{\mathrm{Sn},\mathrm{Ta}:\mathrm{Va}}^{\mathrm{bcc}}=79,927.2$	[14]
	${\;}^{0}L_{\mathrm{Ta},\mathrm{Ti}:\mathrm{Va}}^{\mathrm{bcc}}=12,000$	[12]
	${\;}^{1}L_{\mathrm{Ta},\mathrm{Ti}:\mathrm{Va}}^{\mathrm{bcc}}=-2500$	[12]
	${\;}^{0}L_{\mathrm{Sn},\mathrm{Ti}:\mathrm{Va}}^{\mathrm{bcc}}=-142,089+28.1423T$	[13]
	${\;}^{1}L_{\mathrm{Sn},\mathrm{Ti}:\mathrm{Va}}^{\mathrm{bcc}}=41,211$	[13]
	${\;}^{0}L_{\mathrm{Sn},\mathrm{Ta},\mathrm{Ti}:\mathrm{Va}}^{\mathrm{bcc}}=-400,000$	This work
	${\;}^{1}L_{\mathrm{Sn},\mathrm{Ta},\mathrm{Ti}:\mathrm{Va}}^{\mathrm{bcc}}=-500,000$	This work
	${\;}^{2}L_{\mathrm{Sn},\mathrm{Ta},\mathrm{Ti}:\mathrm{Va}}^{\mathrm{bcc}}=-150,000$	This work
hcp: (Sn,Ta,Ti)_1_(Va)_0.5_	${\;}^{0}L_{\mathrm{Ta},\mathrm{Ti}:\mathrm{Va}}^{\mathrm{hcp}}=8500$	[12]
	${\;}^{0}L_{\mathrm{Sn},\mathrm{Ti}:\mathrm{Va}}^{\mathrm{hcp}}=-127,549.6+23.2048T$	[13]
	${\;}^{1}L_{\mathrm{Sn},\mathrm{Ti}:\mathrm{Va}}^{\mathrm{hcp}}=64,500.5+7.7566T$	[13]
	${\;}^{2}L_{\mathrm{Sn},\mathrm{Ti}:\mathrm{Va}}^{\mathrm{hcp}}=31,287.5$	[13]
bct: (Sn,Ta,Ti)_1_	${\;}^{0}L_{\mathrm{Sn},\mathrm{Ti}}^{\mathrm{bct}}=50,000$	[13]
Ti_3_Sn: (Ta,Ti)_3_(Sn,Va)_1_	${\;}^{0}G_{\mathrm{Ti}:\mathrm{Sn}}^{{\mathrm{Ti}}_{3}\mathrm{Sn}}=-141,133+1.1272T+3\mathrm{GHSERTI}+\mathrm{GHSERSN}$	[13]
	${\;}^{0}G_{\mathrm{Ti}:\mathrm{Va}}^{{\mathrm{Ti}}_{3}\mathrm{Sn}}=15,000+3\mathrm{GHSERTI}$	[13]
	${\;}^{0}G_{\mathrm{Ta}:\mathrm{Sn}}^{{\mathrm{Ti}}_{3}\mathrm{Sn}}=20,000+3\mathrm{GHSERTA}+\mathrm{GHSERSN}$	This work
	${\;}^{0}G_{\mathrm{Ta}:\mathrm{Va}}^{{\mathrm{Ti}}_{3}\mathrm{Sn}}=15,000+3\mathrm{GHSERTA}$	This work
	${\;}^{0}G_{\mathrm{Ta},\mathrm{Ti}:\mathrm{Sn}}^{{\mathrm{Ti}}_{3}\mathrm{Sn}}=-113,207+65T$	This work
Ti_2_Sn: (Ta,Ti,Va)_2_(Sn,Va)_1_	${\;}^{0}G_{\mathrm{Ti}:\mathrm{Sn}}^{{\mathrm{Ti}}_{2}\mathrm{Sn}}=-122,344+6.0034T+2\mathrm{GHSERTI}+\mathrm{GHSERSN}$	[13]
	${\;}^{0}G_{\mathrm{Ti}:\mathrm{Va}}^{{\mathrm{Ti}}_{2}\mathrm{Sn}}=10,000+2\mathrm{GHSERTI}$	[13]
	${\;}^{0}G_{\mathrm{Va}:\mathrm{Sn}}^{{\mathrm{Ti}}_{2}\mathrm{Sn}}=5000+\mathrm{GHSERSN}$	[13]
	${\;}^{0}G_{\mathrm{Va}:\mathrm{Va}}^{{\mathrm{Ti}}_{2}\mathrm{Sn}}=300,000$	[13]
	${\;}^{0}G_{\mathrm{Ti}:\mathrm{Sn},\mathrm{Va}}^{{\mathrm{Ti}}_{2}\mathrm{Sn}}=-34,085.17$	[13]
	${\;}^{0}G_{\mathrm{Ti},\mathrm{Va}:\mathrm{Sn}}^{{\mathrm{Ti}}_{2}\mathrm{Sn}}=-49,803.91+24.471T$	[13]
	${\;}^{0}G_{\mathrm{Ta}:\mathrm{Sn}}^{{\mathrm{Ti}}_{2}\mathrm{Sn}}=15,000+2\mathrm{GHSERTA}+\mathrm{GHSERSN}$	This work
	${\;}^{0}G_{\mathrm{Ta}:\mathrm{Va}}^{{\mathrm{Ti}}_{2}\mathrm{Sn}}=15,000+2\mathrm{GHSERTA}$	This work
	${\;}^{0}L_{\mathrm{Ta},\mathrm{Ti}:\mathrm{Sn}}^{{\mathrm{Ti}}_{2}\mathrm{Sn}}=-145,816+80T$	This work
Ti_5_Sn_3_: (Ta,Ti)_5_(Sn)_3_	${\;}^{0}G_{\mathrm{Ti}:\mathrm{Sn}}^{{\mathrm{Ti}}_{5}{\mathrm{Sn}}_{3}}=-330,186.45+5.3066T+5\mathrm{GHSERTI}+3\mathrm{GHSERSN}$	[13]
	${\;}^{0}G_{\mathrm{Ta}:\mathrm{Sn}}^{{\mathrm{Ti}}_{5}{\mathrm{Sn}}_{3}}=40,000+5\mathrm{GHSERTA}+3\mathrm{GHSERSN}$	This work
	${\;}^{0}G_{\mathrm{Ta},\mathrm{Ti}:\mathrm{Sn}}^{{\mathrm{Ti}}_{5}{\mathrm{Sn}}_{3}}=-179,785+20T$	This work
Ti_6_Sn_5_: (Ta,Ti)_6_(Sn)_5_	${\;}^{0}G_{\mathrm{Ti}:\mathrm{Sn}}^{{\mathrm{Ti}}_{6}{\mathrm{Sn}}_{5}}=-468,938.25+5.3729T+6\mathrm{GHSERTI}+5\mathrm{GHSERSN}$	[13]
	${\;}^{0}G_{\mathrm{Ta}:\mathrm{Sn}}^{{\mathrm{Ti}}_{6}{\mathrm{Sn}}_{5}}=55,000+6\mathrm{GHSERTA}+5\mathrm{GHSERSN}$	This work
	${\;}^{0}L_{\mathrm{Ta},\mathrm{Ti}:\mathrm{Sn}}^{{\mathrm{Ti}}_{6}{\mathrm{Sn}}_{5}}=-300,774$	This work
Ti_2_Sn_3_: (Ta,Ti)_2_(Sn)_3_	${\;}^{0}G_{\mathrm{Ti}:\mathrm{Sn}}^{{\mathrm{Ti}}_{2}{\mathrm{Sn}}_{3}}=-173,931.7+7.82593T+2\mathrm{GHSERTI}+3\mathrm{GHSERSN}$	[13]
	${\;}^{0}G_{\mathrm{Ta}:\mathrm{Sn}}^{{\mathrm{Ti}}_{2}{\mathrm{Sn}}_{3}}=-25,000+2\mathrm{GHSERTA}+3\mathrm{GHSERSN}$	This work
	${\;}^{0}G_{\mathrm{Ta},\mathrm{Ti}:\mathrm{Sn}}^{{\mathrm{Ti}}_{2}{\mathrm{Sn}}_{3}}=-48,000$	This work
Ta_3_Sn: (Ta,Ti)_3_(Sn)_1_	${\;}^{0}G_{\mathrm{Ta}:\mathrm{Sn}}^{{\mathrm{Ta}}_{3}\mathrm{Sn}}=-67,693.3+9.077T+3\mathrm{GHSERTA}+\mathrm{GHSERSN}$	[14]
	${\;}^{0}G_{\mathrm{Ti}:\mathrm{Sn}}^{{\mathrm{Ta}}_{3}\mathrm{Sn}}=20,000+3\mathrm{GHSERTI}+\mathrm{GHSERSN}$	This work
	${\;}^{0}G_{\mathrm{Ta},\mathrm{Ti}:\mathrm{Sn}}^{{\mathrm{Ta}}_{3}\mathrm{Sn}}=-184,472$	This work
Ta_2_Sn_3_: (Ta)_2_(Sn)_3_	${\;}^{0}G_{\mathrm{Ta}:\mathrm{Sn}}^{{\mathrm{Ta}}_{2}{\mathrm{Sn}}_{3}}=-77,332.2+29.6428T+2\mathrm{GHSERTA}+3\mathrm{GHSERSN}$	[14]
Ti_36_Ta_28_Sn_36_: (Ti)_0.36_(Ta) _0.28_(Sn)_0.36_	${\;}^{0}G_{\mathrm{Ti}:\mathrm{Ta}:\mathrm{Sn}}^{{\mathrm{Ti}}_{36}{\mathrm{Ta}}_{28}{\mathrm{Sn}}_{36}}=-22,135.2-10T+0.36\mathrm{GHSERTI}+0.28\mathrm{GHSERTA}+0.36\mathrm{GHSERSN}$	This work

^★^ All parameters are given in J/(mole of atoms); Temperature (*T*) in K. The Gibbs energies for the pure elements are taken from the compilation of Dinsdale [18]. GHSERXX (XX = SN, TA and TI) are the reference state of Gibbs energies.

## 4. Results and Discussion

**Figure 2:**
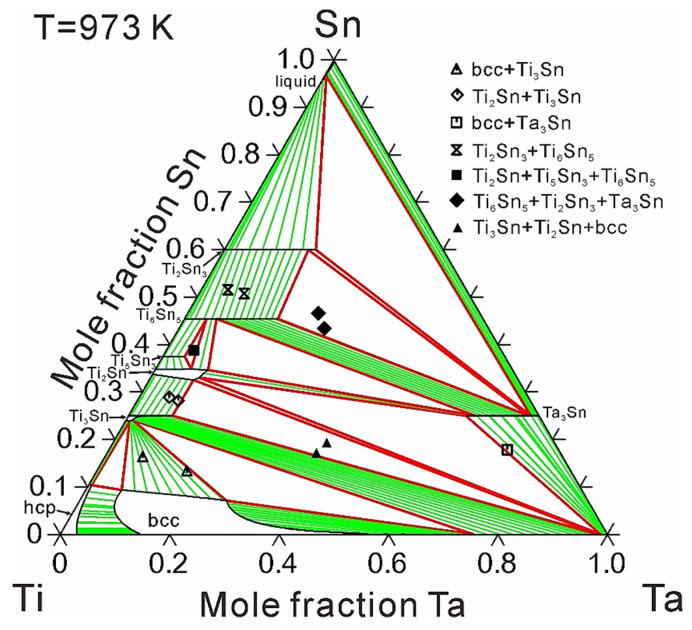
Calculated isothermal section of the Ti-Ta-Sn ternary system at 973 K, compared with experimental data [16].

**Figure 3:**
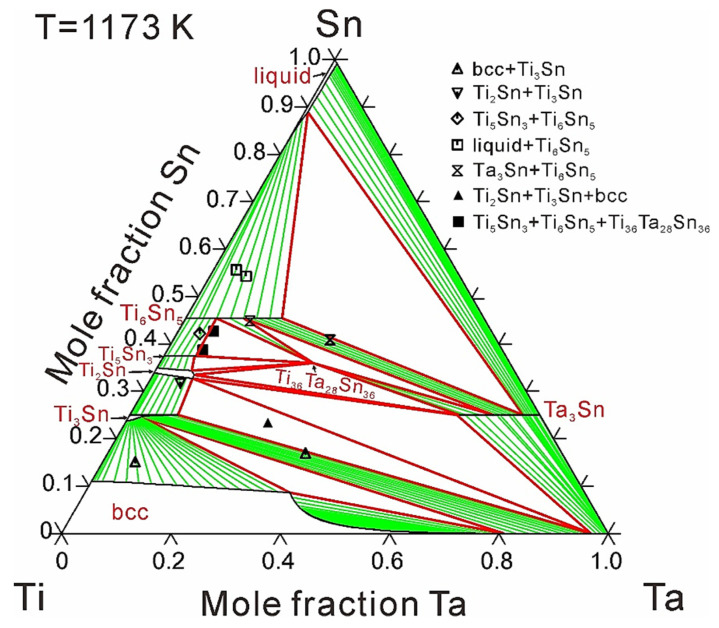
Calculated isothermal section of the Ti-Ta-Sn ternary system at 1173 K, compared with experimental data [17].

Figure 2 and Figure 3 show the calculated isothermal sections of the Ti-Ta-Sn system at 973 and 1173 K compared with experimental information [16,17], respectively. The chemical compositions of the annealed alloys together with the corresponding equilibrium phases are displayed in the figures. Due to small solid solubility or limited experimental verification, ternary solid solubility only considering the substitution between Ti and Ta was employed to describe intermetallic compounds, except Ti_3_Sn and Ti_2_Sn. Table 3 shows the calculated solubilities of the third element in binary compounds compared with experimental data [16,17]. As can be seen, the experimentally determined results can be well reproduced by the present calculations with the consideration of experiment errors.

A total of four two-phase regions and three three-phase regions at 973 K, and five two-phase regions and two three-phase regions at 1173 K have been experimentally detected by Wang et al. [16,17], signed by vacant symbols for two-phase regions and solid symbols for three-phase regions in Figure 2 and Figure 3. As can be seen in Figure 2 and Figure 3, all the three-phase regions and most of the two-phase regions can be well reproduced based on the present thermodynamic modeling. As can be seen, a miscibility gap occurs in the bcc phase. The ternary solid solubility of bcc phase is large at Ti-rich region while rare at Nb-rich region, almost no Sn solubility at the compositions near 50 at.% Ta at 973 K and 65 at.% Ta at 1173 K. This phenomenon may be caused by the over-stability of bcc phase at the Ti-Ta boundary near the Ta corner. Any attempt to enlarge the ternary solubility of the bcc phase near the Ta corner would cause unreasonable miscibility gap of bcc phase far away from the Ti-Ta boundary. As a result, the experimental data about several two-phase regions concerning the bcc phase were given a small weight during the optimization process in order to achieve a general satisfaction. According to the experimental data, the ternary compound, Ti_36_Ta_28_Sn_36_, is stable at 1173 K, while disappears at 973 K. As can be seen, the thermal stabilities of Ti_36_Ta_28_Sn_36_ can be well reproduced by using the present thermodynamic modeling.

**Table 3:** Solubilities of the third element in binary compounds.

Compounds	Temperature, K	Solubilities of the Third Element, at.%		Reference
		Ti	Ta	
Ti_3_Sn	973	-	8.3	[16]
		-	8.0	This work
	1173	-	9.3	[17]
		-	9.0	This work
Ti_2_Sn	973	-	over 8.8	[16]
		-	9.7	This work
	1173	-	over 7.3	[17]
		-	7.6	This work
Ti_5_Sn_3_	973	-	4.2	[16]
		-	4.0	This work
	1173	-	6.1	[17]
		-	6.0	This work
Ti_6_Sn_5_	973	-	16.8	[16]
		-	17.0	This work
	1173	-	over 15.5	[17]
		-	17.6	This work
Ta_3_Sn	973	21.3	over 10.9	[16]
		20.8	11.6	This work
	1173	24.9	over 8.8	[17]
		25.3	15.2	This work

**Figure 4:**
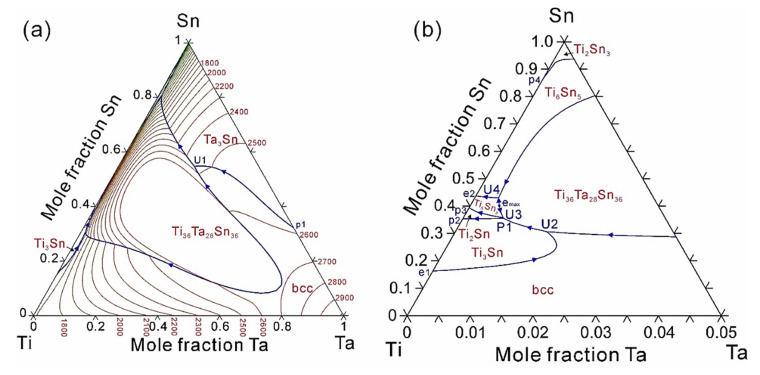
(**a**,**b**) Caculated liquidus projection of the Ti-Ta-Sn ternary system.

On the basis of the presently obtained thermodynamic description of the Ti-Ta-Sn system, the liquidus projection appended with isothermal lines is shown in Figure 4. Figure 4b presents an enlarged region in order to display the complicate reactions near the Ti-Sn boundary, 0 to 5 at.% Ta. The invariant reactions on the liquidus surface are listed in Table 4. As can be seen in Figure 4, for a Ti-Sn alloy, the bcc primary phase region can be significantly enlarged from 16 at.% Sn up to a maximum 30 at.% Sn by doping about 1.5 at.% Ta. Further increasing the content of Ta, the bcc primary phase region shrinks gradually. When doping a large amount of Ta and Sn in Ti-based alloys, Ti_36_Ta_28_Sn_36_ may precipitate as the primary phase instead of bcc phase, which would strongly influence the properties of Ti-based alloys.

**Table 4:** Calculated the invariant reactions on the liquidus surface in the Ti–Ta–Sn system.

Type	Invariant Reaction	Temperature, °C	Source
p1	liquid + bcc = Ta_3_Sn	2582	[14]
U_1_	liquid + bcc = Ta_3_Sn + Ti_36_Ta_28_Sn_36_	2450	This work
U_2_	liquid + bcc = Ti_3_Sn + Ti_36_Ta_28_Sn_36_	1628	This work
e1	liquid = bcc + Ti_3_Sn	1566	[13]
p2	liquid + Ti_3_Sn = Ti_2_Sn	1549	[13]
P2	liquid + Ti_3_Sn + Ti_36_Ta_28_Sn_36_ = Ti_2_Sn	1542	This work
U3	liquid + Ti_36_Ta_28_Sn_36_ = Ti_2_Sn + Ti_5_Sn_3_	1541	This work
U4	liquid + Ti_36_Ta_28_Sn_36_ = Ti_6_Sn_5_ + Ti_5_Sn_3_	1519	This work
p3	liquid + Ti_2_Sn = Ti_5_Sn_3_	1515	[13]
e2	liquid = Ti_5_Sn_3_ + Ti_6_Sn_5_	1488	[13]
p4	liquid + Ti_6_Sn_5_ = Ti_2_Sn_3_	752	[13]

**Figure 5:**
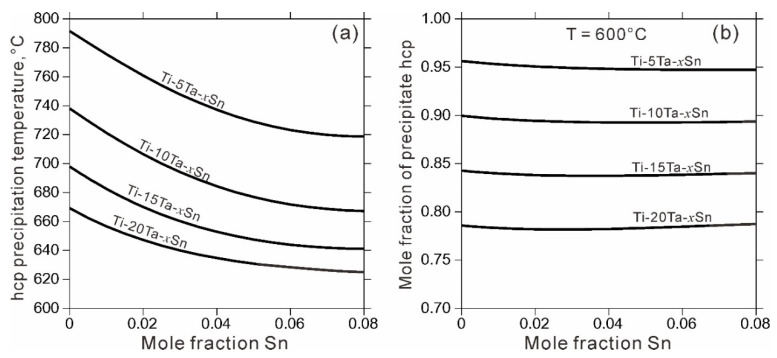
Influence of Ta and Sn contents (at.%) on the precipitation temperature (**a**) and amount (**b**) of α_hcp-Ti phase in β_bcc-(Ti,Ta) phase.

Commonly, Ti based alloys undergo aging treatment after cooling in order to precipitate α_hcp-Ti phase in β_bcc-(Ti,Ta), which is an effective way to improve the mechanical properties. Consequently, it is interesting and technically important to know when the transformation from β_bcc-(Ti,Ta) to α_hcp-Ti takes place and how the alloying element influences the transformation. Figure 5 shows the influence of Ta and Sn content on the precipitation temperature and amount of α_hcp-Ti phase in β_bcc-(Ti,Ta) phase. According to the calculations, it is obvious that increasing the contents of Ta and Sn, especially Ta content, can significantly decrease the transformation temperature. Besides, when aging at 600 °C, the increase in Ta content may reduce the amount of α_hcp-Ti precipitates, while the influence of Sn content is negligible. Thus, prior to experiment, the alloying composition and aging heat treatment schedule can be designed and optimized based on thermodynamic calculations to further obtain optimal properties.

**Figure 6:**
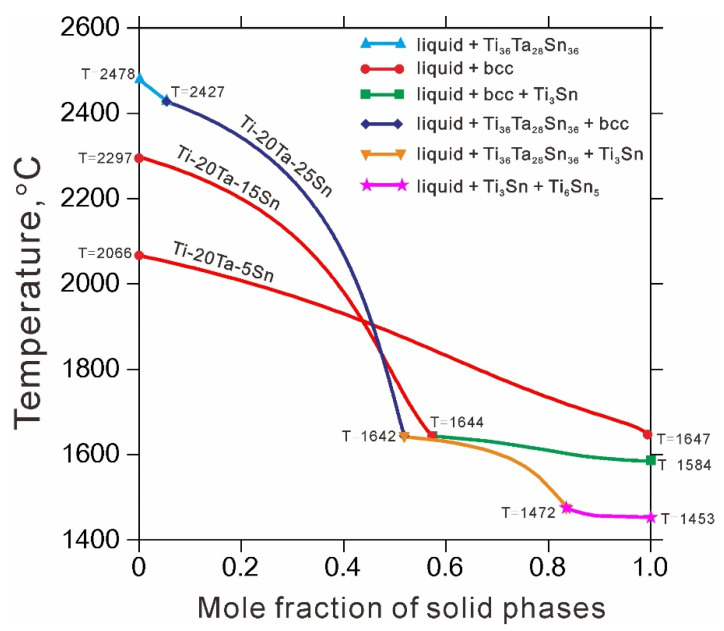
Scheil solidification simulations of Ti-20Ta-*x*Sn (*x* = 5, 15 and 25) (at.%) as-cast alloys.

Recently, Ti-based alloys mainly with the contents of Ta and Sn between 10 and 30 at.% have attracted more and more attentions due to their favorable properties [3,7,28]. Based on the presently obtained thermodynamic description of the Ti-Ta-Sn system, the Scheil solidification simulations of Ti-20Ta-*x*Sn (*x* = 5, 15 and 25) as-cast alloys were performed, as shown in Figure 6. The Scheil solidification curves can provide various information like primary phase, transformation temperature, solidification path and so on. For a Ti-20Ta alloy, when the content of alloying element Sn is less than 25 at.%, the primary phase would be β_bcc-(Ti,Ta) phase. While increasing Sn content up to 25 at.%, the primary phase changes to be Ti_36_Ta_28_Sn_36_. Moreover, large Sn content (exceeding 15 at.%) leads to the formation of Ti_3_Sn and Ti_6_Sn_5_. As can be seen, performing Scheil solidification simulations based on the presently obtained thermodynamic description of the Ti-Ta-Sn system can predict the primary phase and solidified phased in the as-cast alloys, which may affect the subsequent microstructure and properties.

## 5. Conclusions

The phase equilibrium data in the Ti-Ta-Sn system have been critically reviewed. A thermodynamic modeling of the Ti-Ta-Sn system has been performed based on reliable experimental data. Comparisons between experimental and calculated isothermal sections at 973 and 1173 K show that most of the experimental data can be well accounted for by the present thermodynamic description. Based on thermodynamic calculations, the influence of the content of alloying elements Ta and Sn on the precipitation temperature and amount of α_hcp-Ti in β_bcc-(Ti,Ta) and the microstructures of as-cast alloys have been studied, which provides an effective way to design Ti based alloys with high performances.

## Figures and Tables

**Table 1:** Crystal structure data of phases in the ternary system.

Phase	Designation	Prototype	Pearson Symbol	Space Group
α-Ti	hcp	Mg	hP2	P6_3_/mmc
β-Ti	bcc	W	cI2	Im3m
β-Ta	bcc	W	cI2	Im3m
Ti_3_Sn	Ti_3_Sn	Ni_3_Sn	hP8	P4_1_2_1_2
β-Sn	bct	β-Sn	-	I4_1_/amd
α-Sn	diamond	C(diamond)	cF8	Fd3m
Ti_2_Sn	Ti_2_Sn	Ni_2_In	hP6	P6_3_/mmc
Ti_5_Sn_3_	Ti_5_Sn_3_	Mn_5_Si_3_	hP16	P6_3_/mcm
Ti_6_Sn_5_	Ti_6_Sn_5_	Ti_6_Sn_5_	oI44	Immm
Ti_2_Sn_3_	Ti_2_Sn_3_	-	oC40	Cmca
Ta_3_Sn	Ta_3_Sn	Cr_3_Si	cP8	Pm3n
Ta_2_Sn_3_	Ta_2_Sn_3_	CuMg_2_	oF48	Fddd

## Data Availability

Data sharing is not applicable for this article.
